# The Effect of Chitosan on the Chemical Structure, Morphology, and Selected Properties of Polyurethane/Chitosan Composites

**DOI:** 10.3390/polym12051205

**Published:** 2020-05-25

**Authors:** Agnieszka Piotrowska-Kirschling, Joanna Brzeska

**Affiliations:** Department of Commodity Industrial Science and Chemistry, Faculty of Entrepreneurship and Quality Science, Gdynia Maritime University, 83 Morska Street, 81-225 Gdynia, Poland

**Keywords:** chitosan, polyurethane, composites, morphology, crystallinity, thermal properties, sorption, degradability, biological activity

## Abstract

Materials science is an interdisciplinary area of studies. This science focuses on the influence of the physico-chemical properties of materials on their application in human everyday lives. The materials’ synthesis should be developed in accordance with sustainable development. Polyurethanes (PUR) represent a significant consumption of plastic in the world. Modification of PUR, e.g., with polysaccharide of natural origin (chitosan, Chit), should have a positive effect on their functional properties and degradability in the natural environment. The basic parameters affecting the scope and direction of changes are the size and quantity of the chitosan particles. The impact assessment of chitosan on the chemical structure, morphology, thermal properties, crystallinity, mechanical properties, flammability, water sorption, adsorption properties, degradability, and biological activity of PUR/Chit composites (without other additives) is discussed in this article. To the best of our knowledge, recent literature does not contain a study discussing the direct impact of the presence of chitosan in the structure of PUR/Chit composite on its properties, regardless of the intended uses. This paper provides an overview of publications, which presents the results of a study on the effect of adding chitosan in polyurethane/chitosan composites without other additives on the properties of polyurethane.

## 1. Introduction

Polysaccharides are polymers of long chains of monosaccharide units linked via glycosidic bonds [1]. Chitosan (Chit) is a derivative of natural amino polysaccharide and is well described in the literature [1,2]. Chitin is mainly produced from aquatic organism, e.g., shells of shrimps, crustacean, crabs, krill, crayfish, and others [2]. Chit is often obtained by chemical, or sometimes by enzymatic, deacetylation of chitin [2]. It has the linear structure, which is built of glucosamine and *N*-acetyl glucosamine units linked via β(1–4)–glycosidic bonds [1]. Nontoxicity, biocompatibility, biodegradability, antibacterial and antifungal activity [1,2], and ability for adsorption of metal ions, dyes, or organic compounds characterize this polysaccharide [3]. In view of these properties, chitosan materials are mainly used in biomedicine as drug delivery, cosmeceuticals, or pharmaceuticals; in the food industry as packaging paper; in the agriculture industry and in environmental protection as absorber for air and wastewater treatment [2]. Chit can be used for preparation of composites for all the above-mentioned applications [4].

Polyurethanes (PUR) are polymers that have found application in areas such as building, construction (as house insulator or as fire-retardant materials), transportation, automotive industry (in tires, in varying automobiles, and in a number of interior automobile components), electronics (as protection from environmental and mechanical factors), furniture (in cushions and mattresses), cleaning products (sponges), packaging (in products and foods), textiles (as spandex fibers, as a shape-recovery material), footwear (mainly in soles), medicine (as catheters, wound dressings, hydrogels, gloves), and others [5,6]. This versatility of applications means that the amount of polyurethane produced in the world is huge. At present, polyurethane waste is a big problem, the solution of which requires taking extensive measures. Adding a natural, biodegradable material, e.g., chitosan, into the polyurethane network is one of the methods to solve this problem. In addition, such a product has been found to have beneficial properties for many applications. These properties have often proved to be better than pure polyurethane.

The newly obtained materials must be the best possible quality (in terms of functional features) and their production should be economical and environmentally friendly.

**Figure 1:**
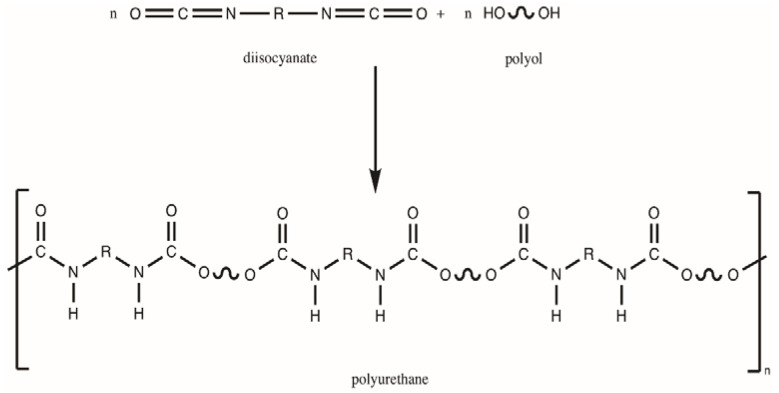
Scheme of preparation of polyurethane.

Polyurethanes are mainly built with isocyanates and polyols (mostly polyethers or polyesters) [5]. A urethane group (–NHCOO–) is created in reaction of isocyanate (–NCO) and hydroxyl groups (–OH), according to the scheme in Figure 1.

PUR composites are characterized by low viscosity, so that they properly combine with various substrates [5]. PUR composites have a wide variety of applications in building [7,8], machine [8], packaging [9], textile [10], and footwear [11] industries, as well as specific applications, e.g., in tissue engineering [12], as drug carriers [13] and sorbents of water and air pollutants [14,15].

In the literature there are known studies about preparing the waterborne polyurethanes based on chitosan [16,17,18,19,20], polyurethanes coated and impregnated via chitosan [21,22,23], polyurethanes with grafted chitosan as a side chain [24,25,26,27,28], and the polyurethane/chitosan composites (PUR/Chit) [29,30,31,32,33,34,35,36,37,38,39,40,41,42,43,44,45,46,47,48]. PUR modification with Chit may have a beneficial effect on the use of these materials and their environmental friendliness.

**Figure 2:**
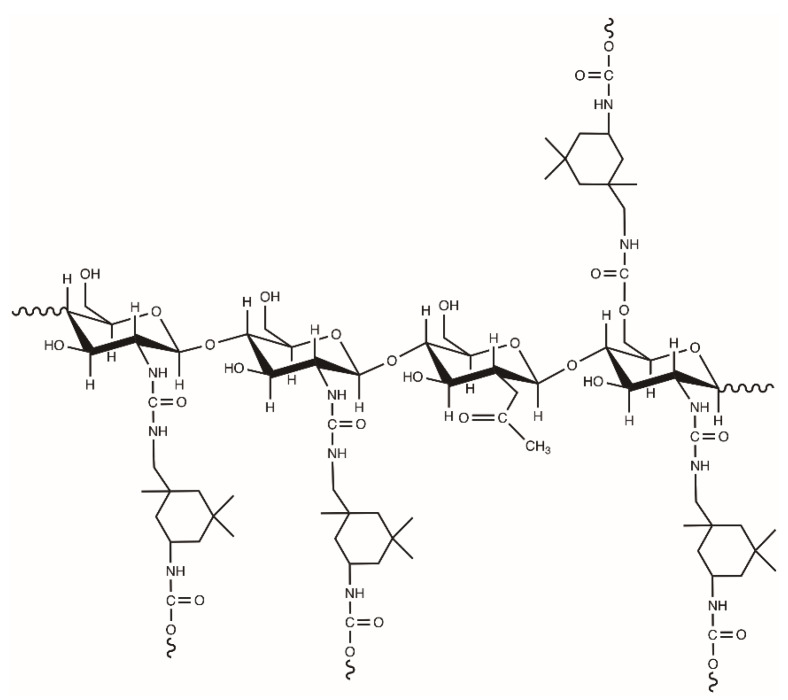
Proposal structure of waterborne polyurethane extended by chitosan (prepared in [20]).

Chitosan can be introduced into waterborne PUR chemical structure [18,19]. Due to the highest reactivity of amine with isocyanate groups, among all functional groups in reaction mixture, Lee and coworkers proposed the processing of waterborne polyurethane synthesis reaction with the formation of urea bonds (Figure 2) [20]. 

Also, Qin et al. showed that, in the case of polyurethane foam synthesis, after introducing an aqueous solution of chitosan into the reaction mixture, while maintaining appropriate conditions, a reaction between the –NCO and –NH_2_ groups occurred [47].

Zia et al. suggested that an appropriate reduction of Chit chains, such as due to H_2_O_2_ treatment, allowed introducing chitosan into chains of PUR elastomers [49]. Other authors stated that chitosan particles can be introduced into the polyurethane structure, claiming that neat chitosan in particles’ forms can react with the isocyanate by –OH and –NH_2_ groups; whereas the low molecular weight of the obtained polyurethanes suggested rather the formation of the PUR/Chit composites with hydrogen-bonded chitosan particles (as indicated by FTIR and NMR results) [50].

**Figure 3:**
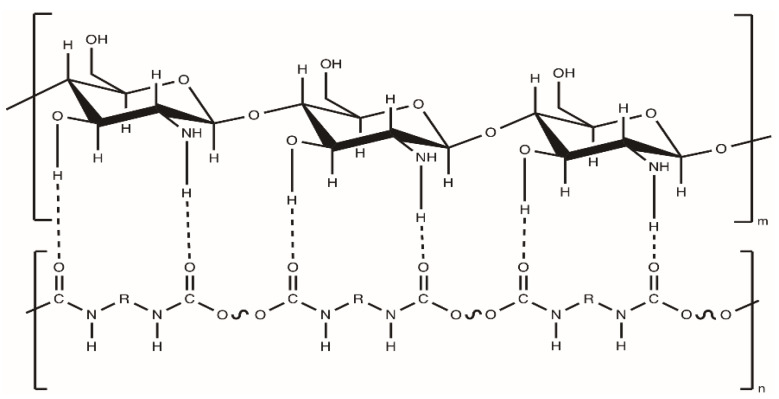
Scheme of proposals of hydrogen interactions between the polyurethane chain and a chitosan particle.

However, the subject of the following work is polyurethane/chitosan composites. Due to the hydrophilic nature of chitosan and the rather low hydrophilicity of polyurethane, both these polymers are, therefore, dissolved in various solvents. Thus, in composites, covalent bonds cannot be expected between them, but only hydrogen bonds’ interactions (Figure 3) [20].

Presence of chitosan particles in polyurethane network influences chains’ ordering and interaction. Consequently, the properties of the resulting composite are changed in comparison to pure polyurethane. The extent and direction of these changes depend largely on the amount of added chitosan particles, their size, and the strength of their interaction with polyurethane chains [29,30,31,32,33,34,35,36,37,38,39,40,41,42,43,44,45,46,47,48]. Depending on these factors, the following phenomena may occur in the structure of the composite: (1) Hindering of the movement of matrix chains; (2) increased cavitation; (3) defects, e.g., holes around filler particles, which, for example, impede the transmission of stress; and (4) particles’ agglomeration. 

The amount of added chitosan in discussed composites varies from 2.5 wt.% to 75 wt.%, whereas the range of chitosan particles’ size is 0.2–12 μm [29,30,31,32,33,34,35,36,37,38,39,40,41,42,43,44,45,46,47,48].

PUR/Chit composites are also often additionally modified with various additives, such as by adding: Starch [50], curcumin [51], β–tricalcium phosphate [52], zinc oxide nanoparticles [41], anatase titania [53], carbon nanotubes [54], graphene oxide [55], polyvinylpyrrolidone [56], and others. Systems for medical purposes are particularly extended with various additions [52,57,58,59]. Such materials for medical applications have been described by Usman et al. [60]. 

However, the purpose of this work was to discuss the effect of adding only chitosan particles into a polyurethane matrix on selected parameters. Further improvements of composites through the use of additional modifiers are extremely interesting. However, in our opinion, the key is to recognize the basic mechanisms and relationships occurring in the polyurethane–chitosan system.

Thus, the impact assessment of chitosan on the chemical structure, morphology, thermal properties, crystallinity, mechanical properties, flammability, water sorption, adsorption properties, degradability, and biological activity of PUR/Chit composites without other additives was the aim of this article. 

## 2. Methods for Determining the Chemical Structure, Morphology, and Selected Properties of Polyurethane/Chitosan Composites

Methods and methodology for determining chemical structure, morphology, thermal properties, crystallinity, mechanical properties, water sorption, adsorption properties, degradability, and biological activity of the studied composites are discussed in this subsection. 

The chemical structure of polyurethane/chitosan composites is determined by a spectroscopic method. The most commonly used method is Fourier-transform infrared spectroscopy (FTIR) [5]. FTIR is also used with attenuated total reflectance (ATR), which is identified as ATR–FTIR. These methods were used to study the chemical structure of PUR/Chit in the literature [31,33,34,35,36,38,40,41,42,44,45,46,47,48].

Morphology of PUR/Chit can be determined by the most commonly used methods, such as microscopy and wide-angle X-ray diffraction analysis (WAXD) [5]. Microscopy is mainly included in optical microscopy (OM) [5], scanning electron microscopy (SEM) [5,61], and atomic force microscopy (AFM) [5,62]. WAXD [29], OM [32,37], SEM [29,30,31,33,35,38,40,42,43,47,48], and AFM [32] were used to determine the morphology of PUR/Chit. These methods allowed us to determine crystallinity/amorphousness, surface topography, roughness [32], porosity, and general morphological structure of the tested materials [29,37].

In the literature, thermal properties of composites are generally investigated using thermal analysis: Thermogravimetry (TGA) [31,33,34,41,44,45,46,47] and differential scanning calorimetry (DSC) [32,34,37]. TGA and DSC often occur as coupled techniques [35]. TGA allows determining thermal stability of materials, whereas, when using DSC analysis, glass transition temperature (*T*_g_), melting temperature (*T*_m_), heat capacity (*C*_p_), and enthalpy of fusion (Δ*H*_m_) can be determined. 

The degree of polymer crystallinity can be determined based on the heat required to melt the polymer by WAXD [5,63,64] or in DSC [5,64,65].

Flammability of PUR/Chit can be studied by limiting oxygen index (LOI), vertical burning test and cone calorimeter test [44,45].

Mechanical properties of the PUR/Chit are usually investigated by a tensile strength instrument [29,34,36,47]. Tensile strength and elongation are determined [36].

Determination of water vapor transport of polymer membranes was studied according to the method which was described by Huang [29,66]. Membranes were investigated by swelling phosphate-buffered saline (PBS) [29,37] e.g., about pH = 7.4 at room temperature [29]. The water sorption capacities were determined by the weight method, taking into account the weight of the tested materials before and after immersing in the medium. Sorption of deionized water of PUR/Chit is also known in the literature [42].

Determination of dye adsorption performance can be studied by using a colorimetric method UV-VIS spectrophotometric (UV-VIS) [30,31,42,48,67,68]. The solution of each dye absorbs visible radiation at the characteristic wavelength [30,42,48]. These studies were investigated by methods which are described in the literature [30,42,48], whereas the adsorption of metal ions can be performed by also a UV-VIS and the Langmuir and Freundlich models [31,67,68]. In the literature, protein adsorption is also known, e.g., bovine serum albumin (BSA) [47]. The samples were incubated in PBS/BSA solution about pH = 7.4 at 37 °C for 3 h.

Degradability of PUR/Chit was studied in hydrolytic, oxidative [32], and in simulated body fluid (SBF) solutions [47]. Changes in chemical structure and thermal properties after degradation were investigated by means of FTIR and DSC.

Antibacterial properties of PUR/Chit were tested by using various kinds of bacteria, which are shown in Table 1 [35,38,39,41]. 

A viability assay can be used for testing a cytotoxic effect of compounds [69]. The influence of PUR/Chit composites on human skin fibroblast (HSF) cells [40] and on osteoblast proliferation and bone mineralization [43] were also discussed.

## 3. Impact of Chitosan on Chemical Structure, Morphology, and Selected Properties of Polyurethane/Chitosan Composites

The effect of adding Chit into PUR composite on the chemical structure, morphology, thermal properties, crystallinity, mechanical properties, flammability, water sorption, adsorption properties, degradability, and biological activity of PUR/Chit composites is described below. Table 2 presents juxtaposition of the discussed PUR/Chit materials. In this table, the used abbreviations of PUR/Chit composites are also presented. Various abbreviations for PUR and Chit are used in the literature. Hence, in this review article, we used the numbering of these materials. Thus, the composites from the first row of Table 2 are named **1. composite**, and so on.

### 3.1. Processing

The addition of solid particles to the bulk polymer or polymer solution is often a big problem when forming a sample into a good-quality composite.

**1. composites** were synthesized by the immersion precipitation phase inversion method [29]. The weight ratio of superfine chitosan powder (SCP) to PUR were 0:100, 10:90, 30:70, 50:50, and 70:30, whereas the average particle size of SCP was 3.28 μm. As the studies showed, the addition of chitosan into PUR bulk accelerated the rate of precipitation of the porous membrane, which was beneficial for its formation. The reason was that water was absorbed by chitosan, which is a hydrophilic material. However, with SCP ratio increasing, the viscosities of blend-casting solutions decreased gradually and, consequently, the looser membrane was formed with more porous and with increased pores’ diameter and samples’ thickness. When the amount of SCP increased to 50 and 70 wt.%, the SCP more strongly aggregated and SCP plastic deformation was observed in the membranes during drying process. It caused the pore collapse and the obtained membranes had low porosities and small pore sizes. Therefore, with SCP:PUR ratio increasing, pore size and porosity firstly increased and then reduced. In addition, analyzing WAXD results, the authors found that the SCP aggregated structure was not destroyed during the processing.

An interesting way to obtain fibers from the **10. composites** (with the weight ratio of 5:95, 10:90, 15:85, and 20:80) was presented by Kang and coworkers [38]. The essence was to find a solvent system that dissolved both components. Using the mixture of 1,1,1,3,3,3-hexafluoro-2-propanol and formic acid as solvents for Chit and PUR dissolution, the authors obtained well-formed fibers via electrospinning method. The more chitosan was introduced into the solution, the thinner were the fibers that were obtained. It was explained by the cationic nature of chitosan that contained the amino groups at the C2 position, which are ionizable under acidic conditions. In consequence, the presence of Chit increased the charge density on the surface of the ejected jet during electrospinning, and higher elongation forces had to be imposed to the jet under the electrical field. It caused increased overall tension in the fibers and the diameter of the final fibers became smaller, which is extremely beneficial.

Similar observations were made by Mohraz et al., who concluded that the optimal parameters for **11. composite** fibers obtained through the electrospinning are: 5% ≤ the weight ratio of Chit in polymeric solution of **11. composites** (%) ≤ 15, 12 ≤ applied voltage (kV) ≤ 17, 10 ≤ tip-to-collector distance (cm) ≤ 18, and 0.3 ≤ polymer flow rate (mL·h^−1^) ≤ 0.7 [39]. The authors used trifluoroacetic acid:dichloromethane solvent system (7:3) and polyurethane:chitosan weight ratios 100:0, 95:5, 90:10, 85:15, and 80:20 for solution preparing.

In contrast, Subramaniam and others used dimethyl fumarate (DMF) as a solvent for obtaining electrospun **12. composites** as membranes. Dissolution of chitosan in DMF was possible due to the size of the chitosan particles used, which were at the nano scale [40]. These authors also observed that the incorporation of Chit nanoparticles into the PUR matrix led to reduction of the fiber diameter.

### 3.2. Chemical Structure

The formation of hydrogen bonds between polyurethane chains and chitosan functional groups is often observed. This is important in terms of increasing the interaction between the two components.

The FTIR spectra of the tested composites confirmed the presence of the characteristic absorption bands for PUR and Chit [31,33,34,35,36,38,40,41,42,44,45,46,47,48]. Adding Chit into PUR matrix caused increasing the band of –O–H and –N–H stretching vibration at about 3500 cm^-1^ – 3400 cm^-1^ and shifting the C=O (from PUR) wave number toward lower values (from about 1700 cm^-1^ to 1640 cm^-1^). These clearly indicated a formation of hydrogen bonds between PUR and Chit in **3. composite** [31].

The similar shifting of carbonyl band to the lower wavenumber (from 1732 cm^−1^ to 1662 cm^−1^) after increasing of Chit in composites was seen on FTIR scans, shown in Figure 1 in publication [34]. The authors obtained and investigated the **6. composites** with 5%, 7.5%, 10%, and 20% flakes of chitosan (M). These flakes were presented in the polymer matrix as homogeneously nanoflakes.

The presence of similar functional groups in **5. composite** made it difficult to follow the changes in the PUR structure after its blending with Chit [33]. However, Arévalo-Alquichire et. all noted that the peak of urea around 1660 cm^−1^ was not formed on FTIR spectra, which means that no secondary reaction was carried out and chitosan was not reacted by covalent bonds with isocyanate.

**Figure 4:**
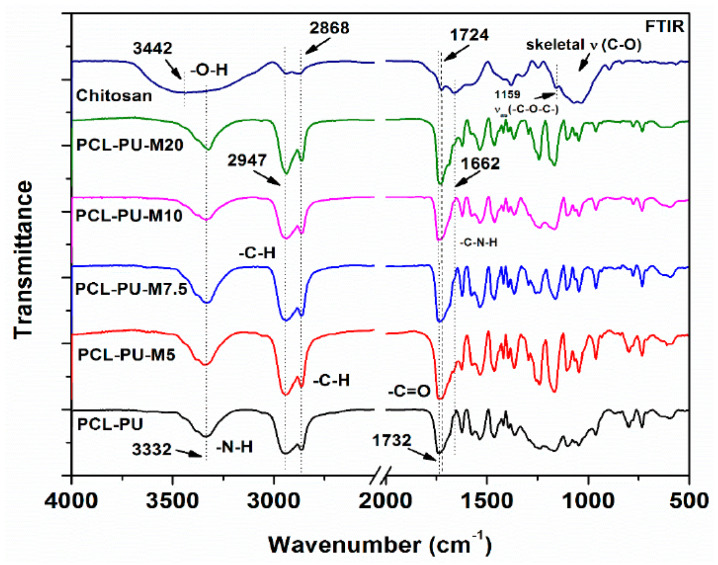
FTIR spectra of chitosan, **6. composites**: polycaprolactone-polyurethane (PCL-PU) and PCL-PU-M (authors called PUR as PU and Chit as M) [34].

Gupta and Kim confirmed by FTIR that after reducing the size of the chitosan to the nano scale, it is possible to introduce chitosan into the polyurethane structure (Figure 4) [34]. After all, they called the obtained materials biocomposites, despite the fact that there is no clear continuous phase (matrix) and filler. However, the trend of changes in the properties of such material are different from conventional composites. The authors point out, e.g., an increase in the mechanical strength of the material after the introduction of chitosan into the polyurethane structure, through an increase in cross-linking.

Using ATR-FTIR for investigation of **8. composites** indicated that Chit particles were immersed inside the matrix bulk, whereas on the surface of samples there were the PUR chains [36].

However, in the case of **13. composites** prepared with PUR and powdered Chit in weight proportion 0.25:0.75, because of a high quantity of Chit, ATR-FTIR spectra showed peaks characteristic for both compounds, which indicated their miscibility [41]. It was also shown that the –NH peak of PU at 3354 cm^−1^ and –OH stretching band of Chit at 3325 cm^−1^ merged and gave a broad band at 3368 cm^−1^, which indicated the formation of hydrogen bonds between PUR and Chit. Also, the peak of stretching vibration of free carbonyl in PUR (1776 cm^−1^), shifted to a lower wave number (1747 cm^−1^) after blending with Chit, confirmed its hydrogen bonding.

The FTIR results showed that adsorption of malachite green via **19. composite** includes hydrogen bond and acetyl groups.

### 3.3. Morphology

The introduction of solid chitosan particles into the polyurethane matrix network must affect its morphology. As the results of the work on this issue show, in fact, the chitosan presence affected both the porosity of the foams and the roughness of the surface of the samples, as well as the morphology of the resulting composites in general. However, sometimes this influence was negligible and depended on different parameters, such as on the size of the introduced particles.

The authors of the previously mentioned paper [29] noted that the content mass of chitosan had little influence on the obtained PUR/SCP (**1. composite**) porous membranes, which was observed in SEM images. It should be mentioned that super-fine powder chitosan was used here. The cross-section showed that membranes had a cellular structure. All upper surfaces of **1. composite** were dense but SCP:PU ratio increasing caused these surfaces to be coarser. With a small SCP content (0 to 30% weight), the bottom surfaces of membranes had uniform pore distribution. However, under 50% weight of SCP, most of the pores collapsed and, as it was shown before, the pores were smaller.

The **2. composites** with different chitosan content (5 wt.%–20 wt.%) were prepared via a combination of foaming and cross-linking process of PU prepolymer and chitosan in the presence of water and glutaraldehyde [30]. The **2. composites’** foams had well-developed open cell structures. Their surfaces were smooth, which could potentially have a beneficial effect on their adsorption properties. Chit powders, with diameter lower than 45 μm, were uniformly embedded in the PUR matrix without aggregates.

Other foam composite of PUR with Chit was obtained using PUR as the polymer matrix and Chit as a filler, in mass relationship 1:1 (50 wt.% of chitosan) [31]. The average pore size of **3. composite** was 127.92 ± 65 μm. The addition of inulin to this composite resulted in a reduction in pore size (95.36 ± 35.91 μm).

The modified PUR by Chit in **5. composite** and the increase of wt.% of Chit caused the improvement of the phase separation [33]. The authors explained this with the agglomeration of chitosan particles. Adding Chit into polyurethane matrix in **6. composite** caused its surface to become less smooth [34].

**Figure 5:**
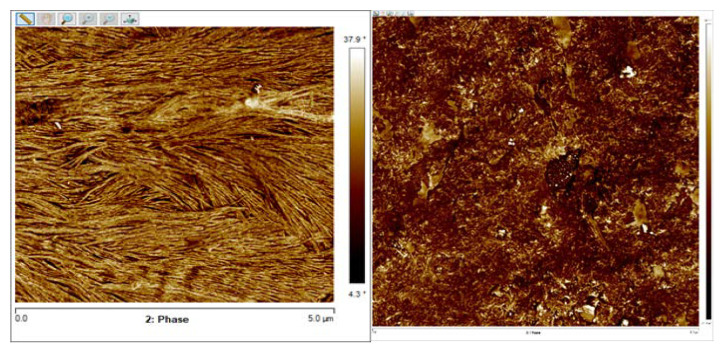
Atomic force microscope images of surface of PUR and its composite with 20 wt.% of Chit (**8. composite**) [36].

Adding Chit into polyurethane matrix in **8. composite** reduced the quantity of nanometric lamellas on the surface of the studied material, as shown in Figure 5.

The reduction of the tendency to form crystalline forms observed in AFM images after adding the chitosan particles into the polyurethane network confirmed DSC results, where the melting enthalpy of the soft segments was found to decrease from 25.8 to 13.1 J·g^−1^ [36].

Any chitosan particles were observed on the surface under optical microscope, which was confirmed by ATR-FTIR and AFM observations that chitosan was deeply embedded in the polyurethane bulk [36]. However, in comparison with pure polyurethanes, the surfaces of their chitosan composites were irregular and much less flat. The authors also examined the addition of montmorillonite into **8. composite,** which caused the surface to become smoother. This might have been due to stronger hydrogen bonds between polyurethane chains and chitosan particles, enhanced by montmorillonite presence.

The morphology of the surface of **9. composites** with 5 and 10 wt.% of chitosan content showed the heterogeneous nature of the tested polymer materials [37]. The optical microscope images of PUR/Chit indicated the influence of chitosan on composite surface in Figure 1 in publication [37].

The authors suggested that the heterogeneity of the surface could be a result of the presence of crystalline domains of soft segments (confirmed by DSC analysis) in the structure of the polymer network. Simultaneously, the heterogeneity of the composite surfaces increased with the quantity of chitosan. Probably the chitosan particles in the PUR with 5% Chit sample were agglomerated, as evidenced by clear “bulges” of the composite surface.

Subramaniam et al. found that the pore size diameter of the electrospun **12. composites** as membranes was reduced compared to pure PUR [40]. This reduction was higher after increasing the quantity of the added Chit. Also, da Rosa Schio et al. found that the addition of Chit into the PUR (**14. composites**) foam resulted in a material with a more developed and opened porous structure [42].

Polyurethane chitosan composites scaffolds, (**15. composites**) obtained by Olivas-Armendariz et al. [43], had high and interconnected pores. This porosity was greater in comparison to pure PUR and the morphology of composites was appropriate for the formation of new tissue via proliferation and differentiation of cells or secretion of its extracellular matrix.

The SEM results showed a well-developed open cell structure of **19. composite,** which had the high porosity (94.44%) [48]. This porosity was similar to pure PUR (95.67%). After adding Chit to PUR, its material porosity did not decrease much. Chit was immobilized in PUR matrix and PUR/Chit had the microporous structure, which was the reason to adsorb malachite green.

### 3.4. Thermal Properties, Crystallinity, and Flammability

Similarly to the structural properties of polyurethane, the presence of solid chitosan particles also influenced the thermal properties. This affected the strength of interactions between polyurethane chains and the possibility of their arrangement, which had a direct impact on the thermal transitions occurring in the sample during its formation and storage. Again, the effect of chitosan on these transformations depended on the size of the chitosan particles.

Medical grade polyetherurethane was used for obtaining **1. composite** [29]. On the WAXD spectrum of PUR a diffraction-wide peak at 20.88° was observed, and the very low degree of crystallinity of pure PUR was found. However, super-fine chitosan powder (SCP) had two diffraction peaks at 10.13° and 19.86°. Adding SCP into the PUR matrix increased its crystallinity, and the intensity of two diffraction peaks for all membranes increased with SCP:PUR ratio. With increasing SCP content, the second diffraction peak shifted to 20.10°. This value was near the PUR diffraction peak. Crystallinity of the samples increased form 5.5% for pure PUR to 33.7% for composite with SCP:PUR = 70:30 ratio.

XRD patters of **14. composite** foam showed that they were a typical semi-crystalline material, with a wide base at 2θ around 20° [42].

According to the Javaid group’s [70], immersing chitosan in a polyurethane structure increased the hydrogen bonds’ length between urethane groups (from PUR) and amine groups (from Chit) and consequently decreased the H– bonding. As a consequence, the move of the soft segments of the PUR in **4. composites** required less energy and *T_g_* was lowered [35].

The **9. composites** of linear polyurethanes with 2.5 wt.% and 5 wt.% chitosan were prepared and investigated [37]. Blending of PUR with Chit increased the crystallinity of PUR soft segments (increased ∆*H*_m_). However, the addition of polycaprolactone triol into the soft segments of similar polyurethanes changed their structure so that blending with chitosan caused a decrease in *T*_g_ and crystallinity (decreased ∆*H*_m_) [35]. Melting of crystalline phase of composites was not observed on DSC thermograms of the second heating cycle.

Nevertheless, in the case of **13. composites** with 75 wt.% of chitosan, *T*_g_ increased and the authors supposed that this was due to the intermolecular hydrogen bonding between PUR and Chit, which reduced chains’ mobility [41]. Shifting the endothermic peak of this composite on DSC thermograms to 152 °C, compared to PUR (108 °C), could have been caused by changes that led to more ordered or aggregated structure in the blended film.

A similar reduction of melting enthalpy of **6. composite** after blending with Chit was observed by Gupta et al. [34]. The 20% weight Chit in this material resulted in the melting temperature of material being higher (increased from 40 °C of polycaprolactone diol to 46.7 °C) and the melting enthalpy was reduced from 23.7 J·g^−1^ to 11 J·g^−1^. The authors explained this by limiting the mobility of polycaprolactone diol (PCL) chains by Chit, and consequently by reducing their ordering and finally crystallinity. The **6. composite** was characterized by high thermal stability, around ~360 °C [34].

The TGA analysis showed the enhancement of thermal stability of PUR with addition of Chit in **7. composites** [35]. All the tested samples had three stages of decompositions. The first stage of decomposition with about 10% weight loss was between 290 and 360 °C and the second stage was between 360 and 420 °C. The samples without Chit were less thermally stable and they had the first stage of degradation, in a range from 240 °C to 309 °C. The second stage also had lower temperature to 367 °C.

The TGA results of **3. composite** showed that they exhibited three stages of decompositions and also had three inflections on the DSC [31]. The first stage of degradation was before 200 °C. The 10% weight loss of **3. composite** was at 280 °C, while 50% was at 430 °C, and the maximum thermal degradation was around 310 °C.

The **5. composite** was thermally stable under 275 °C [33]. This material also had three stages. The 50% weight-loss temperature for these samples (depending on the amount of Chit) was found to be in the range from 381.2 °C to 384.1 °C.

The presence of so many hydroxyl groups in Chit chain caused that it was a potential and promising green charring agent [44,45]. However, the flame retardancy and thermal stability of the material significantly increased after phosphorylation of Chit [46].

The modification of Chit in **16.** and **17.** polyurethane/chitosan **composites** influenced the fire resistance of these materials [44,45]. It can be assumed that the addition of chitosan into polyurethane also improves its flame resistance.

### 3.5. Mechanical Properties

The mechanical testing results for **1. composite** showed that with the increase in the ratio of SCP to PUR, these properties decreased [29]. Tensile strength at break was reduced from 8.19 MPa to 7.71 MPa after adding of 10 wt.% of Chit, and even to 0.71 MPa for composite with 70 wt.% of Chit. Also, elongation at break was reduced. The observed reduction was especially high after increasing the amount of chitosan in composites above 50 wt.%. These reductions destroying the regular and uniform internal structure of the polyurethane and chitosan particles could result in stress convergence. Moreover, the membranes became more and more brittle as the higher quantity of the rigid SCP were added into the composite. Finally, there were observed pores at the interface between the chitosan powder (with μm scale) and polyurethane matrix, which was another reason for the loss in mechanical strength.

However, if introduced into the polyurethane matrix of chitosan having a particle size in the nanometer scale, in an amount up to 10%, it was found that the strength of the composite samples (**6. composite**) increased [34]. Further increasing the amount of chitosan resulted in a significant reduction in mechanical strength. However, **8. composites,** after adding Chit into PUR’ network, had a worse tensile strength than pure PURs, but a bit higher elasticity [36].

Chit with the increase of molecular weight can improve the tensile strength for dry and wet **18. composite** (also in comparison to pure PUR) [47]. Elongation at break had higher values for dry than wet **18. composite**. The increase of molecular weight influenced the increase of wet elongation at break, but in dry it caused a decrease of this property.

### 3.6. Water Sorption and Water Vapor Permeability

It could be said that there was no doubt that the introduction of chitosan into the polyurethane network increased its water sorption properties [29,37,39,42]. This is especially important in the medical applications of these materials.

However, in some cases the hydrophobicity of PUR/Chit composite foam in comparison to pure polyurethane increases. Wang and coworkers found that **18. composites** had lower behavior of the equilibrium water absorption than pure PUR [47]. Furthermore, the authors proved that this hydrophobicity increased with the increase of molecular weight of chitosan from 3000 g·mol^−1^ to 300,000 g·mol^−1^. They stated that high crystallinity and the rigid chain of high molecular chitosan hindered the polar groups to come to the polymer surface.

Much more often, however, it has been found that the addition of chitosan increases the hydrophilicity of the resulting composite. The results of water sorption of polyurethanes and their composites (**9. composites)** showed that adding of 5 wt.% of Chit into the tested composite caused the increase in the amount of absorbed water from 5.5% to 13.9% after 14 days of incubation in deionized water [37].

Much higher quantity of water was absorbed by **14. composite** foam [42]. The swelling degree, estimated after 24 h incubation of the samples in water, increased from 133% for PUR to 162% for composite [42]. The reason was hydrophilicity of chitosan and increased foam porosity.

The presence of super-fine chitosan powder in **1. composite** caused an increase in the water vapor transmission rate [29]. Water vapor permeability occurred in two stages: Adsorption and diffusion, which are connected with composite hydrophilicity and pore morphology of investigated membranes. Both of these parameters were directly affected by the presence of chitosan in the polyurethane network. The authors concluded that as the amount of SCP increased the permeability increased.

### 3.7. Adsorption and Filtration Properties

Very high effectiveness of Chit in adsorption of dyes and heavy metal ions is commonly known [3]. This high efficiency combined with the porous structure of polyurethane foams gives materials with exceptional water-purifying abilities. PUR/Chit composites’ adsorption properties are compared in Table 3. The authors of the research about adsorption properties discussed the results of the maximum capacity adsorption in different ways, so we were not able to standardize them and compare in the table.

The **2. composites’** adsorption properties to Acid Violet 48 were investigated by Lee and coworkers [30]. They obtained composite foams with well-developed open cell structures, and concluded that dye adsorption capacities of the studied composites were increased with the increment of Chit content in composites. The amine groups were the binding sites for sulfonic ions of acid dyes in aqueous solutions. Moreover, it was concluded that dye adsorption capacities of compounds were found to increase with decreasing the pH value. These observed results were from better chemisorption between protonated amine groups of Chit and sulfonic ions of acid dye in available acidic solutions. The maximum adsorption capacity was about 30 mg·g^−1^ for **2. composites** with 20 wt.% Chit content.

Other dye, Food Red 17, was employed for estimation of adsorption capacity of **14. composite** foam obtained by da Rosa Schio et al. [42]. They concluded that composite foam was able to remove >98% of dye from the solution, whereas pure PUR was able to remove only 40%. The presence of Chit in PUR matrix provided additional –OH and –NH_2_ adsorption sites and caused higher porosity.

**Figure 6:**
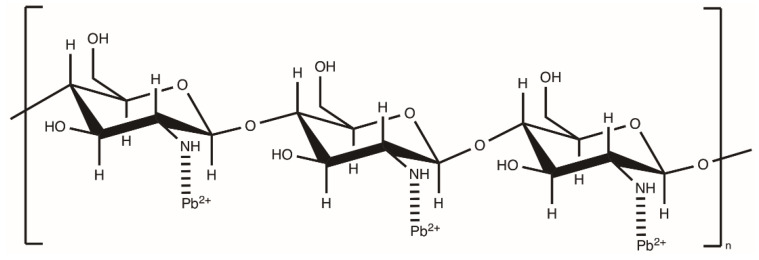
Scheme of the possible interaction between chitosan and Pb(II) (prepared on [31]).

The results of adsorption of **3. composite** showed that adding Chit to PUR improved the adsorption of lead(II) ions [31]. Hernandez-Martinez’s group believed that lead ion was adsorbed by interacting with the nitrogen amino group in Chit (Figure 6). They also concluded that partial replacement of chitosan with inulin caused the increase in adsorption capacity of Pb^2+^, despite that the inulin with PUR was less adsorbent than the tested composite. The authors found that the inulin could replace the Chit in part in the composite, which could reduce production costs.

The **18. composites** had a better adsorbed amount of BSA in comparison with pure PUR [47]. The protein adsorption was improved with the increase of molecular weight of chitosan.

PUR/Chit also was studied for adsorption of the malachite green adsorption (cationic triphenylmethane dye) by the method which is described in the literature [48]. The **19. composite** had a favorable adsorbent for this dye adsorption. Unfortunately, the studied results made by Li did not specify the effect of chitosan concentration on adsorption, but concluded that as the temperature increased, the adsorption efficiency increased, which confirmed that it was due to the chemical adsorption.

The obtained results of **11. composite** as nanofibers’ investigation demonstrated that they provided acceptable filtration performance and could be used in industrial filtration processes (air filters) and personal respiratory protection equipment (face masks) [39].

### 3.8. Degradability

Susceptibility to degradation of the polymeric material is important for its use, e.g., as a carrier of active substances in medicine, cosmetology, and agriculture, as well as due to environmental reasons. Degradability of **4. composites** in hydrolytic and oxidative solutions was estimated by changes in sample mass, thermal properties, and surface morphology (OM and AFM) [32]. The reduction of the sample mass was higher for composites than for pure polyurethanes. The incubation of the samples in degradative solutions influenced the roughness of **4. composites** surface. Before degradation, the surface of materials seemed to be smoother, while after the incubation there were observed cracks. The DSC results of tested composites in both incubated conditions showed that melting temperature and melting enthalpy were increased. This suggested structural changes in the composites. On the one hand, this could indicate an increase in crystallinity and, on the other, an increase in cross-linking.

Qin et al. found that adding chitosan into PUR composite significantly increased its degradability [47]. Moreover, the authors concluded that with increasing molecular weight of chitosan in **18. composites**, the degradation rate increased from 85% to 98%.

### 3.9. Biological Activity

The positively charged surface of the Chit molecules and negatively charged microbial cell membranes are the main reasons for the antimicrobial activity of Chit.

The antibacterial activity increased with concentration of Chit in **7. composite** compared to unmodified PUR [35]. The same relationship was observed for **10. composite** [38].

Mohraz et al. observed the influence of pure PUR nanofibers and **11. composite** (85:15, 15 w/v% (weight/volume percentage concentration)) nanofibers on *Escherichia coli* growth [39]. They concluded that there was no inhibition zone around PUR samples and clear inhibition of bacteria growth around composite sample.

It was also found that the electrospun **12. composites** as membranes showed enhanced attachment and proliferation percent of human skin fibroblast cells in comparison to the pristine PUR membrane [40].

## 4. Conclusions

In the literature, there are known polyurethanes with a variety of properties. Polyurethane modification with chitosan affects its properties and consequently influences their applications. The changes occurring under the influence of chitosan in the polyurethanes structure, which then affect their physico-chemical properties, result from the specific structure of chitosan having many hydroxyl groups. The basic parameter affecting the scope and direction of changes is the size of the chitosan particles. Most authors agree that hydrogen bonds, not covalent ones, form between the added chitosan solid particles and the polyurethane chains in composites. However, by reducing the particle size to nanoscale, it could be expected that NH_2_ groups will begin to react with isocyanate groups and chitosan will be introduced into the polyurethane structure. This kind of material is also called by authors as composite. However, the trends in the properties of such material after using Chit in PUR synthesis are different from conventional composites. In the case of foams and water-borne polyurethanes, after dissolving chitosan in an aqueous acid solution, urea groups between chitosan and diisocyanate are expected.

The chitosan added into the polyurethane matrix very often improves the processing of composites. Usually the crystallinity of PUR/Chit composites is lower than pure PUR. These materials are characterized by three stages of decompositions but different thermal stabilities. The presence of chitosan in polyurethane composites accelerates their degradation in incubation media, which makes them more environmentally friendly. Moreover, the adsorption properties, useful for water treatment, are significantly increased, as is water sorption. Mechanical properties of PUR/Chit composites are mostly lower than pure PUR. However, in some cases, when small amounts of chitosan are used, they may increase. Due to the specific structure of chitosan, its polyurethane composites are characterized by high bioactivity and biocompatibility, which are important in medical applications.

## Figures and Tables

**Table 1:** Antibacterial activity of discussed polyurethane/chitosan composites.

Tested Bacterial	Bacterium Gram Positive (+) Negative (−)	References
*Staphylococcus aureus*	+	[35]
*Bacillus subtilis*	+	
*Escherichia coli*	−	
*Pasteurella multocida*	−	
*Escherichia coli*	−	[38,39]
*Staphylococcus aureus*	+	[41]
*Escherichia coli*	−	

**Table 2:** Juxtaposition of discussed PUR/Chit composites.

Composite No.	Source Abbreviation	Determined Selected Properties										Reference
		Morphology	Chemical Structure	Thermal Properties	Crystallinity	Mechanical Properties	Flammability	Water sorption and Water Vapor Transport	Adsorption and Filtration Properties	Degradability	Biological Activity	
**1.**	SCP/PU	X			X	X		X				[29]
**2.**	PU/chitosan	X							X			[30]
**3.**	PC	X	X	X					X			[31]
**4.**	PUR/Ch	X		X	X					X		[32]
**5.**	PU-PI-CS	X	X	X								[33]
**6.**	PCL-PU-M		X	X	X	X						[34]
**7.**	ANF2-5	X	X	X							X	[35]
**8.**	PUR+Ch	X	X	X	X	X						[36]
**9.**	PUR + 2.5 Ch PUR + 5 Ch	X		X	X			X				[37]
**10.**	chitosan/PU	X	X								X	[38]
**11.**	PU/CH								X		X	[39]
**12.**	SET A SET B	X	X								X	[40]
**13.**	PC1-3		X	X							X	[41]
**14.**	PU/chitosan	X	X					X	X			[42]
**15.**	PU-CH	X									X	[43]
**16.**	TPU-CS	X	X	X			X					[44]
**17.**	TPU/APP/CS	X	X	X			X					[45]
**18.**	PU-CS	X	X	X	X	X		X	X	X		[47]
**19.**	PU/CS	X	X						X			[48]

**Table 3:** Adsorption properties of PUR/Chit composites.

Composite No.	Adsorption Properties		Reference
	Towards	Adsorbate Name	
**2.**	Dye	Acid Violet 48	[30]
**3.**	Cation Metal	Lead(II)	[31]
**14.**	Dye	Food Red 17	[42]
**18.**	Protein	BSA	[47]
**19.**	Dye	Malachite green	[48]
